# Anti-Obesity Effect of Ginkgo Vinegar, a Fermented Product of Ginkgo Seed Coat, in Mice Fed a High-Fat Diet and 3T3-L1 Preadipocyte Cells

**DOI:** 10.3390/nu12010230

**Published:** 2020-01-16

**Authors:** Shugo Hosoda, Yumi Kawazoe, Toshikazu Shiba, Satoshi Numazawa, Atsufumi Manabe

**Affiliations:** 1 Division of Aesthetic Dentistry and Clinical Cariology, Department of Conservative Dentistry, Showa University School of Dentistry, Ohta-ku, Tokyo 145-0062, Japan; hosoda@dent.showa-u.ac.jp (S.H.); shiba@regenetiss.com (T.S.); amanabe@dent.showa-u.ac.jp (A.M.); 2 RegeneTiss Inc., Okaya, Nagano 394-0046, Japan; kawazoe@regenetiss.com; 3 Division of Toxicology, Department of Pharmacology, Toxicology and Therapeutics, Showa University School of Pharmacy, Shinagawa-ku, Tokyo 142-8555, Japan; 4 Pharmacological Research Center, Showa University, Shinagawa-ku, Tokyo 142-8555, Japan

**Keywords:** ginkgo vinegar, *Ginkgo biloba* extract, anti-obesity effect, 3T3-L1 cells, adipocytes

## Abstract

Ginkgo seed coat is rarely used and is typically discarded, due to its offensive odor and its toxicity. Ginkgo vinegar is a fermented product of ginkgo seed coat, and fermentation removes the bad smell and most of the toxicity. Thus, ginkgo vinegar contains very low concentrations of toxic components. The present study examined the anti-obesity effect of ginkgo vinegar in mice fed a high-fat diet and its inhibition of adipogenesis in 3T3-L1 cells. Ginkgo vinegar suppressed high-fat diet-induced body weight gain and reduced the size of fat cells in mice. Ginkgo vinegar suppressed the expression of C/EBPδ and PPARγ, key proteins in adipogenesis, and inhibited lipid accumulation in 3T3-L1 cells that were induced to become adipocytes. These results suggested that ginkgo vinegar inhibited adipocyte differentiation. On the other hand, a corresponding concentration of acetic acid had significantly less effect on lipid accumulation and virtually no effect on adipogenic gene expression. These results suggested that, similar to *Ginkgo biloba* extract, ginkgo vinegar might prevent and improve adiposity. Therefore, ginkgo seed coat could be a useful material for medicinal ingredients.

## 1. Introduction

*Ginkgo biloba* is often planted on city streets all over the world because it is highly resistant to air pollution, such as car exhaust fumes, and it has excellent fire-resistant characteristics. However, there are continual complaints about the bad odor of ginkgo seed coats, which fall on the street and cause odor pollution. In addition, the outer seed coat of *Ginkgo biloba* contains ginkgolic acid and related substances, which are highly allergenic [1], and excessive intake of ginkgo seeds leads to ginkgotoxin poisoning, which causes tonic clonic spasms, vomiting, and loss of consciousness [2,3]. Therefore, despite the fact that ginkgo seed coat is rich in nutrition, the flesh, which accounts for about 75% to 80% of ginkgo seed coat, is discarded with the seeds [4]. Because *Ginkgo biloba* was designated as an endangered species [5], a better alternative would be to make effective use of the offensive seed coat, rather than planting only male trees to avoid the stink.

In the 1960s, a German pharmaceutical company utilized ginkgo leaves for pharmaceuticals. They demonstrated that *Ginkgo biloba* leaf extracts (GbE) could improve blood circulation, inhibit platelet aggregation, and act as antioxidants [6,7,8,9,10]. It was shown that the active ingredients were flavonoids and terpenoids [11,12]. On the other hand, a fermented product of ginkgo seed coat, called ginkgo vinegar, was shown to contain virtually no ginkgolic acids and no offensive order; in fact, it had a nice aromatic scent [13]. Ginkgo vinegar is expected to contain flavonoids and terpenoids and, therefore, it is likely to show biological effects similar to those observed with ginkgo leaves. In addition, fermentation may produce short-chain fatty acids including acetic acid, which increase energy expenditure and thereby reduce obesity risk [14], and various active metabolites of polyphenols, which possess greater antioxidant activity than the respective parent compound [15]. Therefore, ginkgo vinegar would have more potential to improve metabolic syndrome over GbE. The present study demonstrated, for the first time, that ginkgo vinegar was effective on high-fat diet (HFD)-induced obesity in mice. Further in vitro tests of its anti-obesity effects indicated that ginkgo vinegar inhibited adipocyte differentiation. Based on these results, we concluded that ginkgo vinegar, similar to GbE, might prevent and improve adiposity. Therefore, ginkgo seed coat could be a useful material for medicinal ingredients.

## 2. Materials and Methods

### 2.1. Materials

Ginkgo vinegar was provided by the Ginkgo Vinegar Research Institute, Inc. (Koshigaya, Japan). Ginkgolide B and bilobalide were purchased from Nagara Science Inc. (Gifu, Japan). The acetic acid concentration in ginkgo vinegar was calculated to be 5.0%, determined with ion chromatography (Dionex ICS-5000 with an anion exchange column AS20).

### 2.2. Animals

All animal experiments were conducted according to NIH guidelines for the care and use of laboratory animals, and they were approved by the Showa University Institution Animal Care and Use Committee (Permit Number 26045). Male C57BL/6 (6 weeks old) mice were purchased from Japan SLC Co., Ltd. (Hamamatsu, Japan). They were acclimated to the environment for 1 week with a standard chow diet (F2, Sankyo Labo Service Corp, Tokyo, Japan). Mice were then randomly divided into five groups. Four groups of mice were fed a HFD, where fat comprised 60% of the caloric content (New Brunswick, NJ, USA) and one group were fed a standard chow diet (*n* = 5 per group). Mice were given ad libitum access to food and water, which contained 0%, 2.5%, 5.0%, or 7.5% ginkgo vinegar, for 10 weeks. Mice were weighed twice per week.

### 2.3. Histochemical Analysis

Epididymal adipose tissue was dissected and fixed in 10% neutral buffered formalin solution. The fat tissues were embedded in paraffin and cut into sections. Sections were subjected to hematoxylin/eosin (HE) staining, according to the standard protocol. Histological images of fat tissue were acquired with a microscope (Keyence BZ-8100, Kyoto, Japan). The average area covered by 50-60 adipocytes was calculated with Image J software.

### 2.4. Cell Culture

3T3-L1 cells were obtained from JCRB Cell Bank (Osaka, Japan) and grown in Dulbecco’s modified Eagle medium (DMEM) supplemented with 10% fetal bovine serum (FBS) and antibiotics. Cells (1.0 × 10^4^ cells/well) were grown in a 24-well plate for 2 days until confluent (Day 0). Then, the medium was exchanged with induction medium (MDI; DMEM with 10% FBS, 0.5 mM 3-isobutyl-1-methylxanthine, 1 μM dexamethasone, and 10 μg/mL insulin). After a 2-day incubation, the medium was exchanged with a maintenance medium (DMEM with 10% FBS and 10 μg/mL insulin), and cells were incubated for 6 days.

### 2.5. Evaluation of Cytotoxicity and Differentiation

3T3-L1 cells (4.0 × 10^4^ cells/well) were seeded onto 96-well microplates, allowed to attach for 24 h and treated with ginkgo vinegar for another 24 h. Cytotoxicity was evaluated with the Cell Counting Kit-8 assay (Dojindo, Kumamoto, Japan). To evaluate adipogenic differentiation, cells were fixed with 10% formalin for 10 min and rinsed twice with distilled water. Cells were then washed with 60% isopropanol for 1 min and stained with 3 mg/mL Oil Red O dissolved in isopropanol for 20 min. The stained cells were washed once with 60% isopropanol and twice with PBS. Oil Red O was extracted from stained cells by adding 1 mL of 100% isopropanol and rocking cells gently for 5 min. Isopropanol extracts were transferred to a 96-well plate, and absorbance was measured at 492 nm with a microplate reader.

### 2.6. Western Blotting

3T3-L1 cells were cultured in a 24-well plate, washed with PBS, and harvested with 200 µL of sample buffer (2% SDS, 5% 2-mercaptoethanol, 10% glycerol, 0.0005% bromophenol blue, 62.5 mM Tris, pH 6.8). The samples were electrophoresed on a 10% SDS-polyacrylamide gel, and the proteins were transferred to a PVDF membrane. The membrane was blocked for 1 h with 0.2% I block (Thermo Fisher Scientific Inc, Waltham, MA, USA). The membrane was probed with a primary antibody: anti-PPARγ (1:10,000; E-8, Santa Cruz Biotechnology, Inc., Santa Cruz, CA, USA) or anti-C/EBPδ (1:10,000; C-6, Santa Cruz Biotechnology, Inc. Santa Cruz, CA, USA) overnight at 4 °C. The membrane was washed with 20 mM Tris, pH 7.4, 137 mM NaCl, 3 mM KCl, and 0.1% Tween 20 (TTBS) for 10 min, 5 times, then incubated with an appropriate secondary antibody for 1 h at room temperature. After washing with TTBS for 10 min for 5 times, protein bands were detected with a conventional chemiluminescence system (Amersham Biosciences, Piscataway, NJ, USA). The protein expression levels were analyzed with an ImageQuant LAS 4000 mini (GE, Healthcare, Chicago, IL, USA).

### 2.7. Real-Time PCR

Total RNA was extracted from 3T3-L1 cells with an RNeasy mini kit (Qiagen, Hilden, Germany). Reverse transcription was performed with total RNA (100–200 ng) and the High-Capacity cDNA Reverse Transcription Kit (Applied Biosystems, Foster City, CA, USA). Quantitative PCR was performed on an ABI 7500 Fast thermocycler (Applied Biosystems) with the TaqMan gene expression assay system (C/EBPδ, Mm00786711_s1; PPARγ, Mm00440940_m1).

### 2.8. Statistical Analysis

Statistical analyses were performed with a one-way ANOVA, followed by Tukey’s multiple comparisons test unless otherwise specified.

## 3. Results

### 3.1. Suppressive Effect of Ginkgo Vinegar on Weight Gain in Mice

To test whether mice were unwilling to drink ginkgo vinegar, we measured the consumption of drinking water that contained different concentrations of ginkgo vinegar. We observed that water consumption decreased as the concentration of ginkgo vinegar increased to above 10%. On the other hand, with 7.5% or less ginkgo vinegar, mice showed steady consumption, equivalent to plain water consumption (Figure S1). Drinking-water consumption with ginkgo vinegar did not change significantly for at least a week. Therefore, we used 2.5%, 5.0% and 7.5% ginkgo vinegar in the drinking water in the following in vivo experiments. These ginkgo vinegar concentrations were calculated to be 0.12 mL, 0.25 mL and 0.37 mL as a daily intake, respectively.

**Figure 1:**
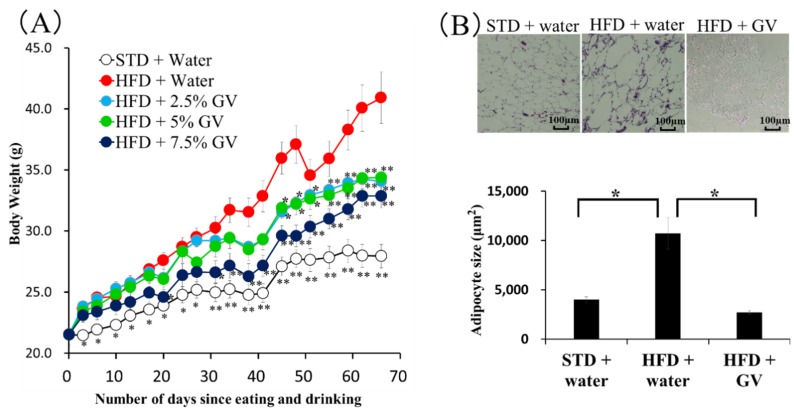
Effect of ginkgo vinegar on high-fat diet (HFD)-induced body fat gain in mice. Mice were fed either standard chow (STD) or a HFD with drinking water that contained 0%, 2.5%, 5.0%, or 7.5% ginkgo vinegar (GV) for 10 weeks. (**A**) Changes in the body weight of mice are illustrated. Data are the mean ± SD (*n* = 5). * *p* < 0.05, ** *p* < 0.01 vs. HFD. (**B**) Sections from epididymal adipose tissues were obtained from mice fed either a STD or HFD with drinking water that contained 0% (plain water) or 5.0% GV for 10 weeks. For quantitative analysis, tissues were stained with hematoxylin/eosin (HE), and the cell areas were evaluated from 4 random fields that contained 50–60 cells. Data represent the mean ± SD (n = 5). * *p* < 0.05.

It was previously reported that GbE attenuated HFD-induced obesity, nonalcoholic fatty liver disease, atherosclerosis, and diabetes mellitus in animals [16,17,18]. To investigate whether ginkgo vinegar had an effect similar to that of GbE on weight gain, mice were fed a HFD and drinking water with 2.5%, 5.0%, or 7.5% ginkgo vinegar. A significant increase in body weight during the whole period of the experiment was observed in the HFD groups compared to the normal diet group. HFD-induced weight gain was significantly suppressed by 7.5% ginkgo vinegar after 31 days, and by 2.5% and 5.0% ginkgo vinegar after 45 days, compared to animals that drank water without ginkgo vinegar. Moreover, ginkgo vinegar suppressed weight gain in HFD-fed mice in a concentration-dependent manner (Figure 1A).

It is widely known that adipose cell size increases during the course of obesity. Therefore, we investigated whether ginkgo vinegar had an effect on the size of fat cells in mice fed a HFD. The HFD significantly increased the size of adipocytes (Figure 1B), measured as the area occupied by adipocytes in HE stained tissues. This HFD effect was nearly completely inhibited by drinking 5.0% ginkgo vinegar (Figure 1B).

### 3.2. Effect of Ginkgo Vinegar on Adipocyte Differentiation In Vitro

The in vivo experiments described above suggested that ginkgo vinegar possessed anti-obesity properties similar to those of GbE [18]. On the other hand, the daily intake of regular vinegar was reported to improve lifestyle-related diseases, including obesity and hyperlipidemia [19]. Therefore, we investigated whether the observed anti-obesity effect of ginkgo vinegar resulted from the acetic acid and other short-chain fatty acids or phytochemicals present in ginkgo seed coat. To address this question, we conducted experiments with 3T3-L1 preadipocytes to determine the effects of ginkgo vinegar on adipocyte differentiation. When various concentrations of ginkgo vinegar were used to test its cytotoxicity on 3T3-L1 cells, we found that 0.8% ginkgo vinegar had a significant effect on cell viability, compared to control culture conditions. In contrast, concentrations of 0.4% or less ginkgo vinegar had no significant effect on cell viability (Figure S2). The acetic acid concentration in ginkgo vinegar was 5.0%, based on ion chromatography. Therefore, we used acetic acid concentrations that corresponded to the amounts of acetic acid in the different ginkgo vinegar conditions to determine cytotoxicity. We found that, in control cultures, 0.04% acetic acid caused a significant difference in cell viability, and 0.02% or less acetic acid had apparently no effect on cell viability (Figure S2). Based on these results, we used 0.4% ginkgo vinegar and 0.02% acetic acid in the following experiments.

**Figure 2:**
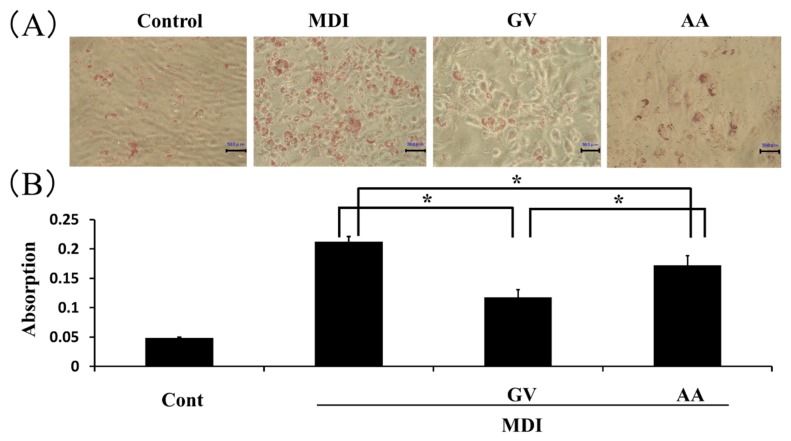
Effect of ginkgo vinegar and acetic acid on lipid accumulation in 3T3-L1 cells. Confluent 3T3-L1 cells were induced to differentiate into adipocytes with differentiation medium (MDI), in the presence of 0.4% ginkgo vinegar (GV) or 0.02% acetic acid (AA) for 5 days. (**A**) Lipid droplets in the cells were stained with Oil-Red O. Scale bar indicates 50 μm. (**B**) The intracellular dye was extracted with 100% isopropanol, and absorbance was measured at 492 nm. Data represent the mean ± SD (*n* = 3). * *p* < 0.05.

To examine whether ginkgo vinegar and acetic acid showed inhibitory effects on adipocyte differentiation, 3T3-L1 cells were treated with 0.4% ginkgo vinegar and 0.02% acetic acid during the course of adipocyte differentiation induced with MDI medium. Both ginkgo vinegar and acetic acid reduced the Oil-Red O-staining of differentiated adipocytes (Figure 2A). However, ginkgo vinegar inhibited adipocyte differentiation more profoundly than the corresponding concentration of acetic acid (Figure 2B).

**Figure 3:**
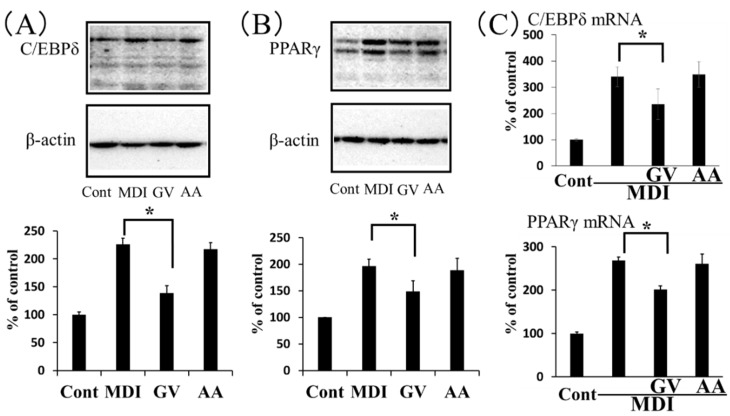
Effects of ginkgo vinegar and acetic acid on the expression of key proteins involved in adipocyte differentiation. Confluent 3T3-L1 cells were induced to differentiate into adipocytes in the presence of 0.4% ginkgo vinegar (GV) or 0.02% acetic acid (AA) for 2 days. (A, B, upper panels) Representative Western blot images show cellular protein levels of (**A**) C/EBPδ and (**B**) PPARγ (lower panels). Results from ImageQuant TL quantifications of band intensities show protein expression levels relative to β-actin expression. (**C**) Real-time PCR results show *C/EBPδ* and *PPARγ* gene expressions in 3T3-L1 cells induced to differentiate into adipocytes in the presence of 0.4% ginkgo vinegar or 0.02% acetic acid. Data represent the mean ± SD (*n* = 3). * *p* < 0.05.

In the early stage of adipogenesis, C/EBPδ, in conjunction with C/EBPβ, induces the expression of C/EBPα and PPARγ, master regulators of adipocyte differentiation [20]. Therefore, we next conducted experiments to determine the effect of ginkgo vinegar on C/EBPδ and PPARγ expression levels in 3T3-L1 cells during the early stage of adipogenesis. We found that, after cells were cultured in MDI medium for 2 days, the C/EBPδ and PPARγ protein levels induced by MDI medium were significantly suppressed by 0.4% ginkgo vinegar. However, the corresponding concentration of acetic acid had apparently no effect on these protein levels (Figure 3A,B). Similarly, ginkgo vinegar significantly inhibited the expression levels of *C/EBPδ* and *PPARγ* genes induced by MDI, but acetic acid appeared to have no effect on the expression of these genes (Figure 3C). These results suggested that ginkgo vinegar specifically suppressed adipogenesis in 3T3-L1 cells.

### 3.3. Effect of Components in Ginkgo Vinegar on Adipogenesis

**Figure 4:**
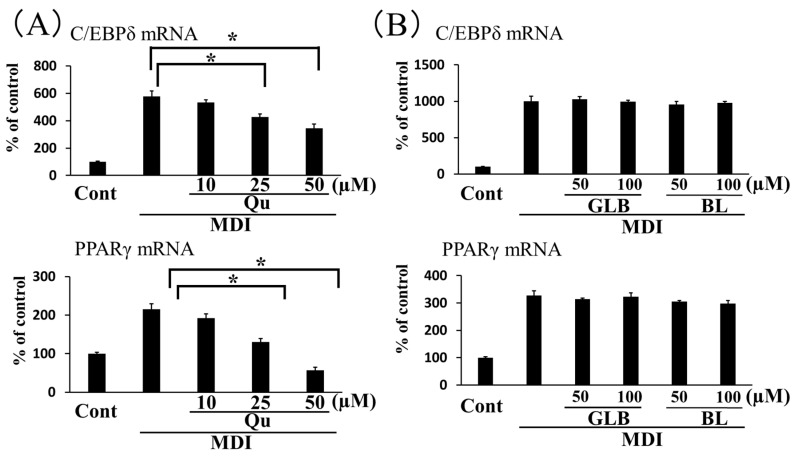
Effects of quercetin (Qu), ginkgolide B (GLB), and bilobalide (BL) on the expression of genes involved in adipocyte differentiation. Qu (**A**), GLB and BL (**B**) were added in conjunction with MDI at the concentrations indicated for 2 days. *C/EBPδ* (upper panel) and *PPARγ* (lower panel) gene expression levels are expressed as the % of control cultures (Cont). Data represent the mean ± SD (*n* = 3). * *p* < 0.05.

GbE was shown to contain flavonoid glycosides, including quercetin, kaempferol, and isorhamnetin, in addition to the terpene lactones, ginkgolide A, ginkgolide B, ginkgolide C, and bilobalide [21]. We reasoned that ginkgo vinegar should have components similar to those found in GbE. Therefore, we examined the effects of quercetin, ginkgolide B, and bilobalide, as representative ginkgo vinegar components, on the expression of genes involved in 3T3-L1 cell adipogenesis. We found that quercetin inhibited C/EBPδ gene expression induced by MDI medium, in a concentration-dependent manner. We also found that quercetin had a more profound inhibitory effect on PPARγ gene expression (Figure 4A). On the other hand, up to 100 μM of ginkgolide B or bilobalide had no effect on the expression of these genes (Figure 4B).

## 4. Discussion

The ability of humans in ancient times to store fat in the body in anticipation of hunger has led to health problems, due to obesity, in modern diets. Obesity is likely to cause diabetes mellitus, hyperlipidemia, hypertension, gout, cholelithiasis, and osteoporosis. Moreover, obesity can contribute to ischemic heart disease, stroke, and myocardial infarction. The present study focused on the ability of ginkgo seed coat to prevent and improve obesity. Ginkgo seed coat is infrequently consumed, due to its malodor and toxicity. Disposal of 75–80% of the ginkgo seed coat produced has raised concern about the environmental impact. On the other hand, components of *Ginkgo biloba* have been shown to have many pharmacological benefits, such as anti-inflammatory effects, positive effects on psychiatric disorders and dementia, and improvements in circulation and diet-induced obesity [6,7,8,9,10,22,23]. Ginkgo vinegar contains flavonoids and terpene lactones, similar to GbE. Polyphenols could be metabolized by the gut microbiota, resulting in the production of several metabolites, which have higher antioxidant activity than the parent compound [15]. Thus, it is possible that fermentation of ginkgo seed coat may produce active flavonoid metabolites. In addition, ginkgo vinegar contains acetic acid and other short-chain fatty acids, which have been shown to reduce obesity risk [14,19]. Therefore, the present study examined the anti-obesity effects of ginkgo vinegar derived from the outer seed coat. We found that ginkgo vinegar suppressed HFD-induced body weight gain and fat cell size in mice (Figure 1). In the present study, mice fed a HFD for 10 weeks did not show alterations in serum markers, including total cholesterol or triglyceride (Table S1). Therefore, at present, it is unknown whether ginkgo vinegar might improve obesity-related diseases. A longer study period is needed to observe in more detail the activity of ginkgo vinegar on diseases derived from obesity.

As mentioned above, acetic acid intake has been shown to improve metabolic symptoms in diabetic rats [24] and obese subjects [19]. Acetic acid concentration in ginkgo vinegar was 5.0%, which accounted for 80% or more of total short-chain fatty acids, as determined by ion chromatography. Therefore, we investigated whether the anti-obesity effect of ginkgo vinegar was merely due to the organic acids present in the vinegar or whether components specifically in ginkgo seed coat played a role. To address this issue, we compared the effects of ginkgo vinegar and acetic acid on the differentiation of 3T3-L1 cells into adipocytes. We found that ginkgo vinegar suppressed the expression of two key proteins in adipogenesis, C/EBPδ and PPARγ. In addition, ginkgo vinegar suppressed lipid accumulation in the cells. We observed similar, but less profound effects with acetic acid added at the concentration that corresponded to the concentration of acetic acid in ginkgo vinegar (Figure 3). These results in conjunction to the previous findings [19,24] suggested that both the components specific to ginkgo seed coat and the organic acids in the vinegar inhibited adipogenesis.

GbE was shown to exhibit a wide range of pharmaceutical activities on obesity-related conditions, including HFD-induced obesity, diabetes mellitus, and non-alcoholic fatty liver disease [16,17,18]. In addition, several flavonoid components of GbE, including quercetin, kaempferol, and isorhamnetin were reported to show beneficial effects on lipid profiles [25,26,27]. The present study also demonstrated that quercetin was effective in inhibition of adipogenic gene expressions during 3T3-L1 differentiation (Figure 4A), with observations consistent with previous findings [28]. Ginkgolides A, B, and C were shown to have wide variety of biological activities, including the inhibition of inflammatory responses, fatty acid synthesis, and lipid accumulation in cells [21,29]. On the other hand, ginkgolide B and bilobalide showed apparently no inhibitory effect on adipogenic gene expressions in our experimental protocol (Figure 4B), where effect on the initial stage of adipogenic differentiation was examined. The previous studies showed the anti-adipogenic effect of these terpenoids on the later stage of differentiation of the preadipocytes [29,30]. It is necessary to examine the effects of ginkgo vinegar in experiments that act on differentiated adipocytes in the coming study.

## 5. Conclusions

The present study demonstrated that ginkgo vinegar reduced the HFD-induced body weight gain of mice, and suggested that at least a part of the effect is related to the suppression of adipocyte differentiation. In addition to the anti-obesity effects, ginkgo vinegar could show many of the pharmacological activities observed with GbE and short-chain fatty acids and, therefore, is expected to be applied to improve metabolic syndromes in the future.

## Supplementary Materials

The following are available online at https://www.mdpi.com/2072-6643/12/1/230/s1. Figure S1: Changes in the consumption of drinking water containing various concentrations of ginkgo vinegar. Mice were given drinking water containing 0%, 2.5%, 5.0%, 7.5%, 10.0%, 12.5% or 15.0% ginkgo vinegar for 3 days. Consumption of the drinking water is illustrated as mL/day/mouse. Data represent mean ± SD (*n* = 3). * *p* < 0.5, Dunnett’s test; Figure S2. Cytotoxicity of various concentrations of ginkgo vinegar (A) and acetic acid (B) on 3T3-L1 cells. 3T3-L1 cells were induced into adipocytes with MDI medium in the presence of 0.1–0.8% ginkgo vinegar (GV) or 0.005–0.04% acetic acid for 2 days. Cell viability was assessed by trypan blue dye exclusion test. Data represent mean ± SD (*n* = 5). ** *p* < 0.01 vs. MDI; Table S1. Effect of ginkgo vinegar and HFD on serum cholesterol and triglyceride in mice. Mice were fed either normal chow (STD) or HFD with drinking water containing 0 (water), 2.5, 5.0 or 7.5% ginkgo vinegar (GV) for 10 weeks. Serum levels of total cholesterol (T-CHOL) and triglyceride (TG) were analyzed (mg/dL) in Fuji Film VET Systems Co Ltd. (Tokyo Japan). Date represent mean ± SD (*n* = 3–5).
