# Supplementary material for: Anti-Obesity Effect of Ginkgo Vinegar, a Fermented Product of Ginkgo Seed Coat, in Mice Fed a High-Fat Diet and 3T3-L1 Preadipocyte Cells
Source: Nutrients. 2020 Jan 16;12(1):230. doi: 10.3390/nu12010230 (PMC7019924; doi:10.3390/nu12010230)
Supplement: Supplementary file 1 [file nutrients-12-00230-s001.pdf]

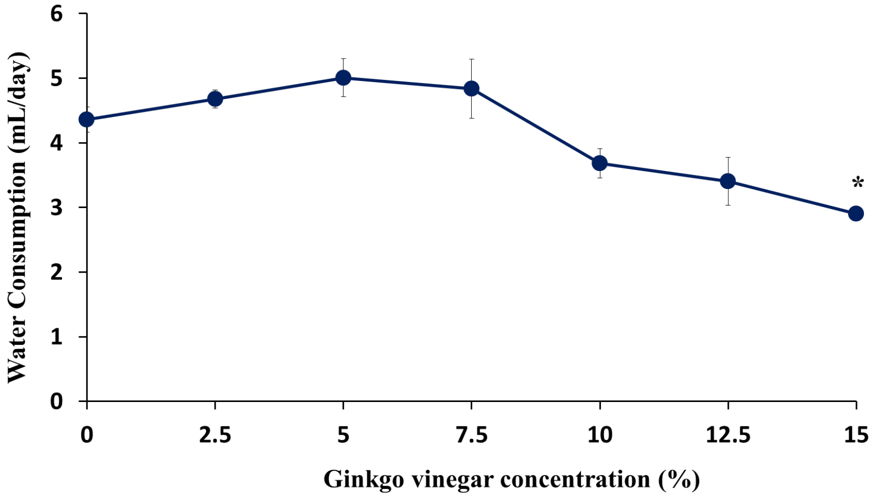

Figure S1. Changes in the consumption of drinking water containing various concentrations of ginkgo vinegar. Mice were given drinking water containing 0%, 2.5%, 5.0%, 7.5%, 10.0%, 12.5% or 15.0% ginkgo vinegar for 3 days. Consumption of the drinking water is illustrated as mL/day/mouse. Data represent mean ± SD ($n = 3$). * $p < 0.5$, Dunnett's test.

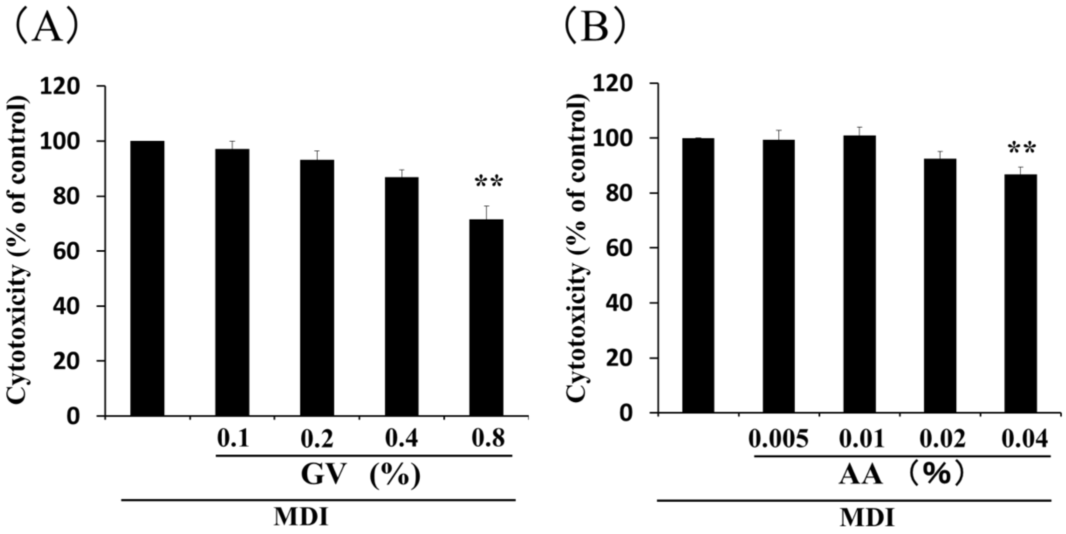

Figure S2. Cytotoxicity of various concentrations of ginkgo vinegar (**A**) and acetic acid (**B**) on 3T3-L1 cells. 3T3-L1 cells were induced into adipocytes with MDI medium in the presence of 0.1%–0.8% ginkgo vinegar (GV) or 0.005%–0.04% acetic acid for 2 days. Cell viability was assessed by trypan blue dye exclusion test. Data represent mean ± SD ($n = 5$). ** $p < 0.01$ vs. MDI.

Table S1. Effect of ginkgo vinegar and HFD on serum cholesterol and triglyceride in mice.

	STD + Water	HFD + Water	HFD + 2.5% GV	HFD + 5.0% GV	HFD + 7.5% GV
T-CHOL	147.5 ± 11.1	178.2 ± 45.6	149.2 ± 20.3	139.8 ± 45.9	159.1 ± 7.1
TG	27.3 ± 5.3	30.0 ± 8.8	41.2 ± 8.6	33.8 ± 11.9	33.8 ± 22.1

Mice were fed either normal chow (STD) or HFD with drinking water containing 0 (water), 2.5%, 5.0% or 7.5% ginkgo vinegar (GV) for 10 weeks. Serum levels of total cholesterol (T-CHOL) and triglyceride (TG) were analyzed (mg/dL) in Fuji Film VET Systems Co Ltd. (Tokyo, Japan). Date represent mean ± SD (n = 3–5).
